# Individualization of clinical target volume delineation in eccentric nasopharyngeal carcinoma: a prospective comparative study

**DOI:** 10.3389/fonc.2025.1587764

**Published:** 2025-08-04

**Authors:** Yunrui Song, Yuwei Wang, Mengqi Yang, Xinhao Yu, Mengze Li, Bin Long, Xiaolei Shu, Xin Zhang, Feng Wang, Chencheng Wang, Mengyu Hu, Jiang-Dong Sui, Ying Wang

**Affiliations:** ^1^ Radiation Oncology Center, Chongqing University Cancer Hospital, School of Medicine, Chongqing University, Chongqing, China; ^2^ Department of Oncology, The Fifth People 's Hospital of Chongqing, Chongqing, China

**Keywords:** eccentric NPC, clinical target volume, dosimetry, plan evaluation, radiotherapy techniques

## Abstract

**Background:**

Clinical target volume (CTV) delineation is a major focus in radiotherapy for nasopharyngeal carcinoma (NPC) and currently lacks a universally accepted standard across treatment centers. We proposed an individualized CTV delineation method for eccentric NPC and evaluated its feasibility based on the eccentric distance of the primary lesion.

**Materials and methods:**

Ninety patients with eccentric NPC were included. Each treatment plan was replanned using the individualized CTV method for dosimetric comparison with the conventional CTV, to evaluate coverage, homogeneity, and conformity of CTV and PTV, sparing of organs at risk (OARs) and radiotherapy technique. Paired sample t-tests and nonparametric rank-sum tests were used to compare target coverage, homogeneity, conformity, and OAR dose parameters between the two approaches. Correlation analysis is used to evaluate the correlation between eccentric distance of primary lesion and OARs dose changes. Subgroup analysis is used to compare the PTV and OARs dose parameters of individualized CTV at different T stages or radiotherapy techniques.

**Results:**

Our results showed that compared with conventional CTV, the volume of CTV decreased significantly (P< 0.05) through individualizing delineation for eccentric NPC, especially CTV1 volume (95.81 cm³ vs. 57.57 cm³, P < 0.001). Individualized CTV reduced the doses delivered to OARs, including the brainstem, spinal cord, optic chiasm, optic nerves, and contralateral temporal lobe, inner ear and so on (all P< 0.05). When the eccentric distance of the primary lesion was between 1.4 and 2.1 cm, the individualized CTV approach provided significant advantages in organ protection, such as contralateral optic nerve, temporal lobe and parotid gland. Additionally, Subgroup analysis showed that the dose-sparing benefit of individualized CTV was more pronounced in patients treated with VMAT (volumetric modulated arc therapy).

**Conclusion:**

This study demonstrates the dosimetric advantages of individualized CTV delineation based on eccentric distance. Our prospective trial is currently ongoing for further research (NCT06167109).

## Introduction

Nasopharyngeal Carcinoma (NPC) is a malignant tumor originating from the nasopharynx mucosa, which has a high incidence in the south of China, Southeast Asia, and North Africa. With the application of intensity-modulated radiotherapy (IMRT), the survival rate of NPC has significantly improved, with a 5-year local control rate of 85-90% (1, 2). However, acute complications such as hearing loss (47.4%) and xerostomia (83.0%) (2), and late complications such as temporal lobe injury (13.1%) and cranial nerve injury (14.0%) (3) still seriously affect the quality of life of patients. Complications following radiotherapy are mainly caused by excessive irradiation of the corresponding normal tissue range and dose. Therefore, reducing the toxic and side effects of patients has become a research hotspot recently, and the treatment mode has changed to individualization (4), such as asynchronous chemotherapy for early and low-risk patients, reducing the dose of radiotherapy and narrowing the range of irradiation.

There is still no recognized delineation standard for the delineation of clinical target volume (CTV). Restricted the anatomical structure around the nasopharynx to a certain distance based solely on experience would not meet the requirements of precise treatment (5). As a result, researchers have continued to optimize the CTV for these patients to minimize radiotherapy-related side effects without affecting survival rate. A retrospective study (6) found that simultaneously reducing the volume and dose of CTV did not affect local area control rates, but significantly reduced late-stage adverse reactions, particularly pulmonary infection, dysphagia, and xerostomia. Miao et al. (7) researched on 103 patients with stage I and II NPC. CTV2 was defined as the omission of the posterior third of the maxillary sinus and cavernous sinus, and patients with N0–1 were defined as the omission of the pterygopalatine fossa, lateral pterygoid muscle, post-styloid space, foramen ovale, and spinous process. The study achieved satisfactory 10-year LRFS (90.3%) and overall survival (91.2%). Only 1 patient had local recurrence 6.1 months after radiotherapy, 4 patients (3.9%) had acute grade 3 mucositis, and 1 patient (1.0%) had acute grade 3 radiation dermatitis. These results support the notion that appropriately reducing CTV volume does not impair the disease control rate. However, most studies have ignored the distribution characteristics of tumors when reducing the volume of CTV.

The application of individualized CTV delineation should also consider the location of the lesion. Most NPC patients present eccentricity, accounting for 72.4% of all NPC cases, and it was found that the spread of eccentricity lesions is concentrated in the ipsilateral pharyngeal recess. The ipsilateral invasion rate of most anatomical structures is significantly higher than that of the contralateral side, and the risk of bilateral invasion is low (10%) (8). The extreme typical case of eccentric NPC is unilateral NPC with a nasopharyngeal mass limited to one side of the nasopharynx and not exceeding the midline, which accounts for about 10% of all NPC cases (9). Li et al. (10) performed a pathological examination of contralateral pharyngeal recess (CPR) and contralateral posterior superior wall (CPSW) in 20 patients with unilateral NPC, and analyzed the factors related to contralateral tumor infiltration to explore the potential of reducing CTV. It was found that unilateral NPC rarely had CPR infiltration, so CTV did not include CPR in patients without lateral lymph node metastasis or EBV-DNA negative. According to the trend of tumor invasion, Sanford et al. (11) carried out individualized target delineation for NPC radiotherapy, excluding all traditional high-risk areas of NPC, and for unilateral NPC, excluding the contralateral parapharyngeal space and skull base channel. It was showed a 5-year local control rate of 94% and no recurrence of contralateral tissue structure. The optimization of the contralateral risk structure reduced the dose of the contralateral parotid gland, optic nerve, and cochlea in T1 patients by 54%, 50%, and 34%, respectively, and the dose of the optic chiasm and contralateral optic nerve in T4 patients decreased by 46% and 37%, respectively. Another study has summarized the local invasion characteristics of unilateral NPC through MRI, which is characterized by continuous invasion from the proximal to the distal end. The probability of contralateral parapharyngeal space and skull base channel invasion is very low (12). Therefore, conventional prophylactic irradiation should not be performed on the contralateral side with a low risk of invasion for patients with eccentric NPC, as this would reduce the volume of CTV, alleviate post-radiation complications, and improve the quality of life.

At present, there is little research on individualized treatment for eccentric NPC, as well as its protective effect on OARs. To determine whether our individualized CTV offers an advantage over conventional CTV, We are the first center to compare the dosimetry parameters of PTV and OARs in patients with eccentric NPC and evaluate radiotherapy techniques suitable for individualized CTV.

## Materials and methods

### Patients

From September 2021 to July 2023, 90 patients with newly treated eccentric NPC at Chongqing University Cancer Hospital were enrolled. Other inclusion criteria were: 1) meeting the definition of eccentric nasopharyngeal carcinoma (patients with a nasopharyngeal mass confIned to one side of the nasopharynx or did not exceed the midline.); 2) having no distant organ metastasis; 3) with baseline MRI data of the nasopharynx and neck.

Informed consent was obtained from all participants at baseline and all procedures were performed in accordance with our local guidelines and clinical regulations. The study was conducted in accordance with the Declaration of Helsinki, and the protocol was approved by the Ethics Committee of The Chongqing University Cancer Hospital (No. CZLLYJ0252). Our prospective trial is currently ongoing for further research (NCT06167109, 12/12/2023).

### Clinical target volume delineation and prescription dose

**Figure 1:**
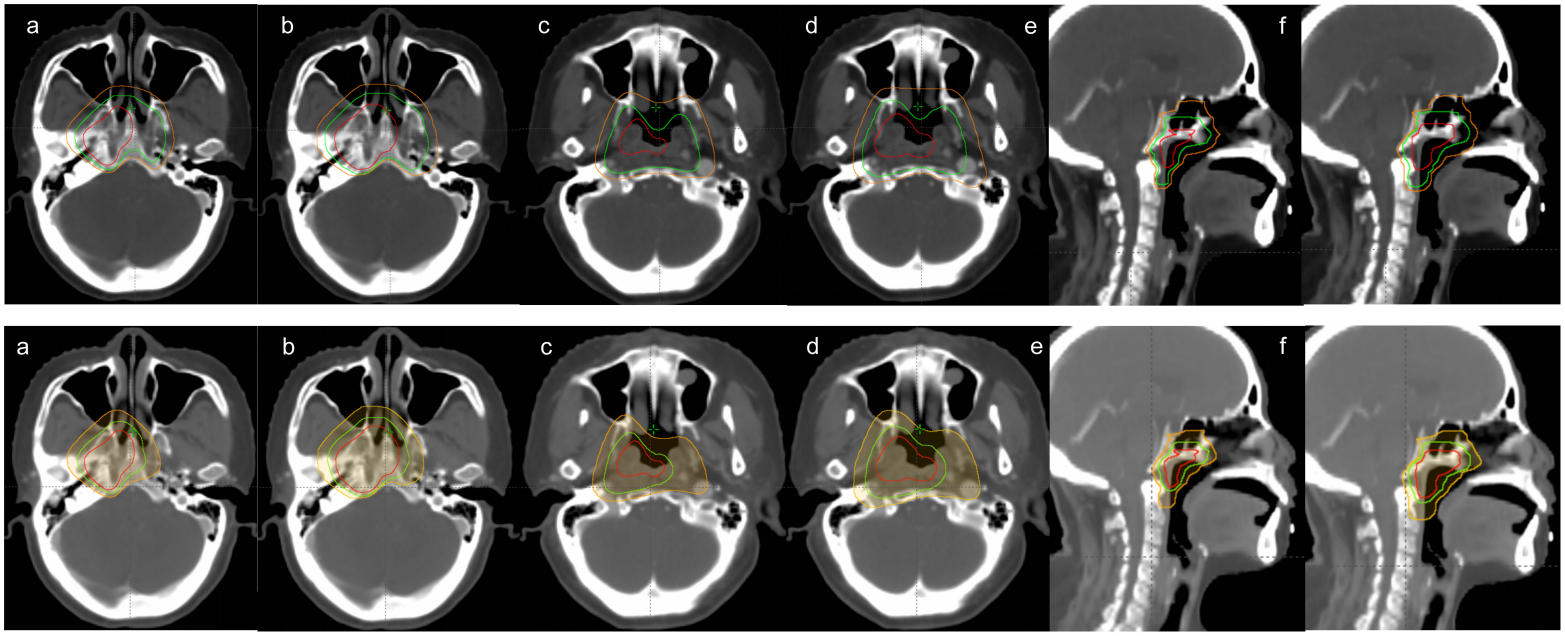
Comparison of conventional(up) and Individualized(down) CTV delineation. The images of **(a)**, **(c)** and **(e)** represent the delineation of the GTV, CTV1 and CTV2, and the images of **(b)**, **(d)** and**(f)** exhibit the delineation of PGTV, PTV1 and PTV2. The GTV and PGTV is in solid red, CTV1 and PTV1 is in solid green, and CTV2 and PTV2 is in solid orange.

We redesigned a new plan based on the individualized CTV delineation, but it was not used for radiotherapy. Gross tumor volume(GTV) is the primary nasopharyngeal lesions, positive retropharyngeal lymph nodes (GTVnx), and cervical lymph nodes (GTVnd) by imaging examination. CTV1 and CTV2 represent high-risk and low-risk invasion areas, respectively. For individualized CTV, the contralateral CTV1 was defined as a subclinical disease consisting of a 5mm margin surrounding GTVnx(not including entire nasopharynx mucosa); the CTV2 was defined as potentially involved regions consisting of a 5mm margin surrounding the CTV1, and the contralateral CTV2 only included the pharyngeal recess. For conventional CTV, the CTV1 is defined as GTVnx+5mm+entire nasopharyngeal mucosa. The CTV2 is defined as the CTV1 + 5mm+corresponding anatomical structures. The delineation of the CTV is outlined in **Table 1** and **Figure 1**.

**Table 1:** The individualized and conventional CTV delineation of eccentric NPC.

Structures	Conventional	Individualized
CTV1	GTVnx+ 5mm+ entire nasopharyngeal mucosa	GTVnx+5mm(not including entire nasopharynx mucosa)
CTV2	CTV1 + 5mm+ corresponding anatomical structures	Ipsilateral: CTV1 + 5mm+ corresponding anatomical structures Contralateral: only included the pharyngeal recess
Nasal cavity	At least 5 mm from choana	At least 5 mm from choana (Ipsilateral side only)
Clivus	1/3 if no invasion; whole if invasion	Cover ipsilateral 1/3 if no invasion; whole if invasion
Maxillary sinus	At least 5mm from maxillary sinuses posterior wall	Cover at least 5mm from ipsilateral maxillary sinuses posterior wall if ipsilateral pterygopalatine fossa invasion; Omitted if not
Ethmoid sinus	Include vomer and the surrounding ethmoid sinus	Omitted if no invasion
Sphenoid sinus	Inferior part in T1-T2, the entire sphenoid sinus in T3-T4;	Ipsilateral: Inferior part in T1-T2, the entire sphenoid sinus in T3-T4; Contralateral: Omitted if no invasion
Skull base	Cover bilateral foramina ovale, rotundum, lacerum, and petrous tip	Only cover Ipsilateral foramina ovale, rotundum, lacerum, and petrous tip
Retropharyngeal lymph nodes	Base of skull to cranial edge of the hyoid	Base of skull to cranial edge of the hyoid if positive Omitted if negative

GTVnx, the primary nasopharyngeal gross tumor volume; CTV, clinical target volume.

The dose prescriptions of PGTVnx, PGTVnd, PTV1, and PTV2 were 69.96-74.2Gy, 69.96-74.2Gy,60.06-60.8Gy and 54.12-54.4Gy in 30-33fractions, respectively. All plans meet the following requirements: 100% of the prescribed dose should cover more than 95% of the target volume, the maximum dose point is located in the target area, and PTV should not receive more than 110% of the prescribed dose. In order to maintain consistency, the delineation of the target volume and OAR was performed by a doctor who specializes in radiotherapy for NPC. The dosimetric restriction of the OARs are shown in **Supplementary Table 1**. For example, the Dmax of the brainstem is 54Gy, the Dmax of the spinal cord is 45Gy, and the Dmean of the parotid gland is 26Gy.

### Plan evaluation

90 patients with eccentric NPC underwent a revised radiotherapy plan, which was completed by senior medical physicists at our center. The Kappa analysis involved two radiation oncologists (with 8+, and 10+ years of experience, respectively), each independently reviewing the same 20 randomly selected cases. The evaluation process included standardized training on contouring criteria (Conventional and Individualized for CTV) prior to analysis. The prescribed doses were consistently maintained and dose and site validation was performed to meet clinical treatment criteria. The plan was evaluated according to the dose-volume histogram(DVH) generated after the optimization of the treatment planning system (TPS) plan.

The IMRT plans were generated in the Eclipse TPS (Version 15.6) based on multi-leaf collimators Millennium 120, and a 6MV X-ray in a Varian IX linear accelerator was used. The coplanar irradiation of 9 fields (200°, 240°, 280°, 320°, 0°, 40°, 80°, 120°, 160°) was used. The VMAT plans were generated in the Pinncle^3^ 16.2 TPS (Version 16.2, Philips, Inc., USA). All plans used double-arcs, with a rotation angle of 181°to 179°and a bed angle of 0°. The TOMO plans were designed using the three-dimensional TPS of the Tomotherapy Planning Station with 6MV X-ray. The dose calculation was performed using the convolution superposition algorithm. The main planning parameters were: field width = 2.512cm, pitch = 0.287 and modulation factor = 2.2-3.5.

Dosimetric validation was performed using a square polymethyl methacrylate (PMMA) phantom with a PTW Freiburg ionization chamber (model TN30013). Measurements followed AAPM TG-51 protocols, with beam calibration at 6 MV (SSD = 100 cm, depth = 5 cm). Prior to clinical implementation, all treatment plans underwent comprehensive 3D dose verification utilizing the ArcCHECK dosimetry system. Clinical approval required fulfillment of pre-established quality assurance criteria, specifically a gamma index passing rate exceeding 95% under 3%/3 mm evaluation parameters.

### PTV coverage

The dosimetric parameters were quantified to evaluate PTVs included V100%, Dmean, D2%, D95%, D98%, heterogeneity index (HI) and the conformity index (CI). V100% is the percentage of the volume of PTV1 and PTV2 covered by prescription isodose of PTV1 and PTV2; D2%, D95%, and D98% are doses that cover 2%, 95%, and 98% of the PTV volume, respectively, with D2% approximating the maximum dose and D98% approximating the minimum dose.

HI was calculated as HI= D5%/D95% to evaluate the homogeneity of the prescription dose in target volume (D5%: the dose received by 5% of the target volume; D95%: the dose received by 95% of the target volume). The smaller the HI, the better the homogeneity. CI was calculated as CI= Vt,ref/Vt×Vt,ref/Vref to evaluate the conformity of the prescription dose in target volume (Vt,ref: the target volume surrounded by the prescription dose; Vt: the target volume; Vref:the volume surrounded by the prescription dose). The ideal CI value is 1, and the closer it is to 1, the better the conformity.

### Organs at risk

The maximum doses(Dmax) of the spinal cord, temporal lobe, optic chiasm, optic nerve, pituitary, lens, inner ear, and brainstem; the mean doses(Dmean) of the parotid gland, thyroid gland, larynx, pharyngeal constrictor, pituitary, oral cavity and inner ear; the D0.03ccPRV of the spinal cord, temporal lobe, optic chiasm, optic nerve and brainstem; and the V30 (the volume that received 30Gy) of parotid gland were analyzed and recorded.

### Statistical analysis

Statistical analysis was performed using SPSS version 27. The descriptive analysis of the data is expressed in terms of means and standard deviations. The dosimetric parameters of the PTV and OAR were assessed and compared using the paired-sample t-test and nonparametric rank-sum test. Correlation analysis was used to evaluate the correlation between eccentric distance of primary lesion and OARs dose changes. Subgroup analysis was used to compare the PTV and OAR dosimetric parameters of individualized CTV at different T stages or radiotherapy techniques. The two-tailed P values < 0.05 are considered statistically significant.

## Results

This study included 90 NPC patients with eccentric lesions. The median patient age was 52 years (24-76). There were 39, 31, and 20 patients in the T1-2, T3, and T4 stages, respectively. Histologically, 85(94%) patients had non-keratinized carcinoma, and 5(6%) had keratinized carcinoma. The number of patients treated with IMRT, VMAT, and TOMO were 20, 34, and 36 respectively. A total of 82 patients received chemotherapy, 65 of whom underwent concurrent chemoradiotherapy. All patients were staged according to the 8th AJCC staging system, and the detailed clinical data of patients are presented in **Table 2**. The Kappa analysis indicated good inter-physician consistency in target volume delineation (**Supplementary Table 2**).

**Table 2:** Clinical characteristics of patients.

Characteristics	N (%)
Median age (range), years	52 (24-76)
Gender	
Male	64 (71.1%)
Female	26 (28.9%)
Site	
Right	49 (54.4%)
Left	41 (45.6%)
Clinical stage	
I	1 (1.1%)
II	21 (23.3%)
III	29 (32.2%)
IVa-b	39 (43.3%)
T classification	
1	2 (2.2%)
2	37 (41.1%)
3	31 (34.4%)
4	20 (22.2%)
N classification	
0	5 (5.6%)
1	41 (45.6%)
2	17 (18.9%)
3	27 (30.0%)
Chemotherapy	
Yes	82 (91.1%)
Induction	3 (3.3%)
Concurrent	14 (15.6%)
Induction + Concurrent	65 (72.2%)
No	8 (8.9%)
Pre-treatment Epstein-Barr virus DNA	
Positive	53 (58.9%)
Negative	36 (40.0%)
Unknown	
Radiotherapy technique	1 (1.1%)
IMRT	20 (22.2%)
VMAT	34 (37.8%)
TOMO	36 (40.0%)

**Figure 2:**
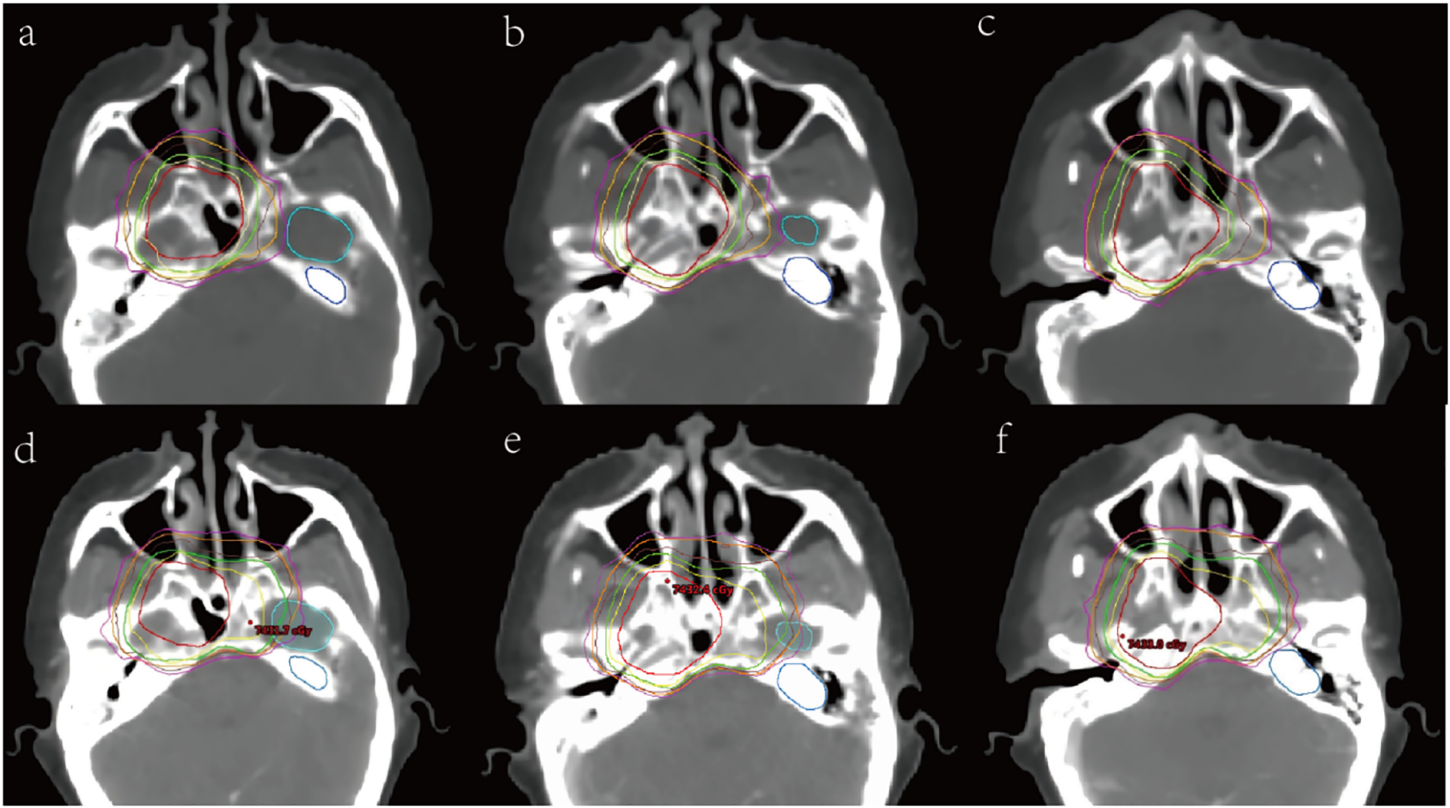
Dose distribution comparison images of conventional (down) and Individualized (up) CTV delineation. **(a)**, **(b)** and **(c)** are individualized cross-sectional dose distribution maps, while **(d)**, **(e)**, and **(f)** are conventional cross-sectional dose distribution maps. The PGTV is in solid red, PTV1 is in solid green, and PTV2 is in solid orange. The 95% dose line of 69.96Gy is yellow, the 95% dose line of 60.06Gy is brown, and the 95% dose line of 54.12Gy is purple. The light blue structure is the left temporal lobe, and the dark blue structure is the left inner ear.

As shown in **Figure 1** and **Supplementary Table 3**, the volume of individualized CTV significantly decreased compared to the conventional CTV (CTV1: 95.81 cm^3^ vs. 57.57 cm^3^, CTV2: 428.50 cm^3^ vs. 395.05 cm^3^; P< 0.001). Strikingly, we found that individualized CTV reduced the doses delivered to OARs generally, with a significant decrease in the D0.03 and Dmax of organs which belong to the priority level one, including the spinal cord, brainstem, optic chiasm, optic nerve, and contralateral temporal lobe (P< 0.05) (**Supplementary Table 4**, **Figure 2**). In addition, an obvious decrease (P< 0.05) was seen in the Dmean of the parotid gland, thyroid gland, pituitary and inner ear, the Dmax of the lens, pituitary, inner ear. However, there was no distinct difference among the doses of the larynx, pharyngeal const and oral cavity (**Supplementary Table 4**). Next, subgroup analysis was performed to compare the specific advantages of the two kinds of delineation schemes to different OARs in different T stages. In the group of stage T1-2, significant differences were observed in D0.03 and Dmax of the spinal cord, optic chiasm, optic nerve, brainstem, contralateral temporal lobe, Dmean of contralateral inner ear, and Dmax of the lens and pituitary (**Table 3**). The individualized CTV for patients in stage T3–4 also experienced a sharp decrease in D0.03 and Dmax of the optic chiasm, optic nerve and contralateral temporal lobe, Dmean of the contralateral inner ear, Dmax of the lens, but not differences of spinal cord, brainstem and pituitary (**Table 3**).

**Table 3:** Comparison of organs at risk in different T stages.

Objective	Index	T1-2(Mean ± SD)		P-value	T3(Mean ± SD)		P-value	T4(Mean ± SD)		P-value
		Conventional	Individualized		Conventional	Individualized		Conventional	Individualized	
Spinal cord	Dmax(cGy)	3256.0 ± 255.6	3140.0 ± 284.3	0.023	3304.1 ± 242.0	3236.9 ± 313.7	0.210	3433.7 ± 510.8	3429.3 ± 569.2	0.970
	D0.03cc(cGy)	3207.4 ± 249.0	3095.7 ± 264.8	0.018	3234.1 ± 222.1	3164.6 ± 279.3	0.176	3324.7 ± 503.6	3330.0 ± 553.2	0.601
Temporal lobes-I	D0.03cc(cGy)	5955.3 ± 331.9	5781.7 ± 643.6	0.049	6549.2 ± 419.0	6380.7 ± 620.5	0.050	6864.8 ± 174.7	6904.4 ± 314.7	0.167
	Dmax(cGy)	6127.0 ± 337.9	5942.5 ± 587.2	0.009	6676.9 ± 412.6	6503.7 ± 599.9	0.042	7009.9 ± 138.9	7003.1 ± 304.2	0.296
Temporal lobes-C	D0.03cc(cGy)	5819.3 ± 296.8	4340.9 ± 731.3	0.000	5847.9 ± 340.3	4365.2 ± 815.1	0.000	6132.3 ± 313.2	4901.3 ± 753.0	0.000
	Dmax(cGy)	5967.3 ± 293.2	4508.6 ± 702.9	0.000	5996.2 ± 340.0	4526.7 ± 807.6	0.000	6308.6 ± 329.2	5086.1 ± 761.4	0.000
Optic chiasm	D0.03cc(cGy)	3427.4 ± 1010.3	2873.2 ± 1038.4	0.000	4925.0 ± 723.9	4268.2 ± 989.9	0.000	5392.5 ± 484.3	5066.4 ± 624.2	0.002
	Dmax(cGy)	3579.4 ± 1033.7	3007.9 ± 1034.7	0.000	5122.0 ± 733.7	4498.1 ± 985.4	0.000	5559.4 ± 525.3	5311.6 ± 611.1	0.007
Optic nerves-I	D0.03cc(cGy)	3386.0 ± 1270.6	2853.7 ± 1330.3	0.000	4998.3 ± 916.8	4316.5 ± 1216.0	0.000	5516.6 ± 695.2	5147.4 ± 1023.4	0.019
	Dmax(cGy)	3556.9 ± 1256.9	2980.6 ± 1329.7	0.000	5180.7 ± 856.4	4521.3 ± 1184.2	0.000	5713.9 ± 771.5	5398.9 ± 1048.6	0.048
Optic nerves-C	D0.03cc(cGy)	3517.9 ± 1300.4	2182.9 ± 1150.3	0.000	4791.6 ± 861.5	3126.3 ± 1034.4	0.000	5088.8 ± 369.9	3483.4 ± 1000.8	0.000
	Dmax(cGy)	3671.1 ± 1308.1	2320.5 ± 1147.6	0.000	5049.9 ± 817.1	3325.2 ± 1049.2	0.000	5270.2 ± 226.3	3736.7 ± 1056.3	0.000
Parotid gland-I	V30(%)	41.8 ± 11.7	41.8 ± 12.1	0.734	42.6 ± 10	38.0 ± 10.1	0.011	50.2 ± 22.6	49.7 ± 23	0.723
	Dmean(cGy)	3072.0 ± 692.5	3021.0 ± 639.3	0.315	3062.2 ± 414.4	2890.4 ± 430.0	0.001	3543.0 ± 1203.2	3489.6 ± 1190.8	0.156
Parotid gland-C	V30(%)	39.1 ± 5.9	39.6 ± 7.3	0.521	41.0 ± 12.1	38.1 ± 14.5	0.033	42.2 ± 7.0	40.4 ± 9.2	0.263
	Dmean(cGy)	2880.5 ± 227.7	2839.8 ± 271.5	0.364	3010.1 ± 564.0	2838.7 ± 642.8	0.002	3063.5 ± 323.1	2957.2 ± 404.8	0.079
Thyroid	Dmean(cGy)	4296.4 ± 650.8	4201.9 ± 650.4	0.004	3988.3 ± 690.2	3924.8 ± 781.5	0.308	4415.9 ± 469.1	4416.1 ± 397.3	0.601
Larynx	Dmean(cGy)	3813.9 ± 276.0	3753.5 ± 292.8	0.308	3726.5 ± 304.2	3715.7 ± 394.6	0.938	3784.2 ± 341.0	3834.1 ± 401.3	0.351
Pharyngeal const	Dmean(cGy)	3941.2 ± 298.2	3960.9 ± 414.9	0.791	3894.7 ± 298.2	3871.6 ± 403.4	0.969	3851.7 ± 375.9	3871.6 ± 410.2	0.455
Pituitary	Dmean(cGy)	4538.7 ± 490.8	4029.8 ± 747.4	0.000	5161.7 ± 478.1	4929.3 ± 590.0	0.010	5636.2 ± 589.8	5444.7 ± 515.0	0.067
	Dmax(cGy)	5212.9 ± 294.6	4895.6 ± 596.9	0.000	5623.7 ± 486.8	5583.6 ± 545.7	0.518	6369.1 ± 490.4	6259.6 ± 527.0	0.218
Oral cavity	Dmean(cGy)	3467.1 ± 246.1	3445.3 ± 313.3	0.586	3467.3 ± 280.1	3455.8 ± 280.9	0.814	3489.1 ± 258.3	3477.5 ± 311.6	0.351
Lens-I	Dmax(cGy)	393.5 ± 121.9	322.3 ± 124.1	0.000	550.9 ± 142.1	479.8 ± 142.1	0.000	654.5 ± 450.4	538.7 ± 147.1	0.207
Lens-C	Dmax(cGy)	393.3 ± 111.4	312.6 ± 112.2	0.000	541.4 ± 126.4	442.8 ± 115.3	0.000	531.3 ± 142.8	500.1 ± 153.5	0.081
Inner ear-I	Dmax(cGy)	5667.8 ± 581.9	5653.8 ± 572.0	0.149	6677.6 ± 795.4	6615.9 ± 845.0	0.033	7153.8 ± 626.4	7207.7 ± 526.9	0.526
	Dmean(cGy)	3656.9 ± 391.1	3564.4 ± 323.5	0.065	4377.6 ± 742.7	4254.9 ± 716.4	0.003	4926.9 ± 659.6	4882.6 ± 581.8	0.575
Inner ear-C	Dmax(cGy)	5423.4 ± 414.5	4587.9 ± 699.7	0.000	5459.6 ± 456.1	4415.4 ± 549.6	0.000	5758.1 ± 405.1	4920.5 ± 519.3	0.000
	Dmean(cGy)	3589.3 ± 355.2	2986.7 ± 432.8	0.000	3716.0 ± 401.7	3027.4 ± 493.1	0.000	3754.5 ± 356.6	3401.0 ± 455.6	0.011
Brain stem	D0.03cc(cGy)	4760.5 ± 290.8	4607.7 ± 288.4	0.008	5126.2 ± 403.4	4871.4 ± 470.8	0.001	5417.8 ± 367.3	5406.9 ± 542.2	0.627
	Dmax(cGy)	4836.2 ± 283.6	4687.8 ± 280.8	0.009	5273.8 ± 427.1	4950.4 ± 491.4	0.000	5610.7 ± 370.8	5484.6 ± 489.1	0.247

I, ipsilateral; C, contralateral.

**Figure 3:**
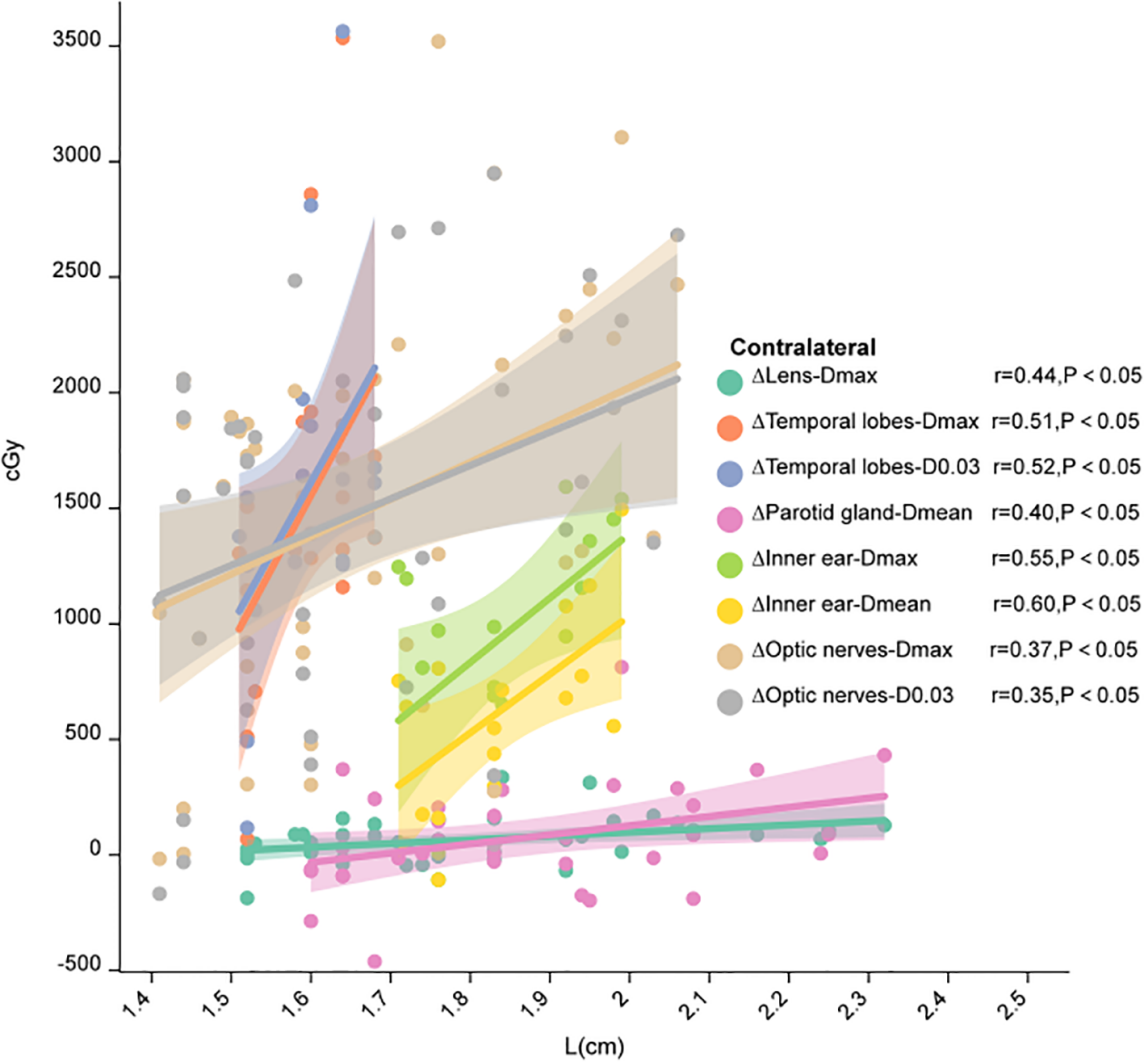
Correlation between L and the dose difference of the contralateral structure. This figure was drawn using ChiPlot (https://www.chiplot.online/) (accessed on May 2024).

The distance from the tumor center to the midline of the body (L for short), which represented the eccentric extent of primary lesion, is positively correlated with the reduction of the CTV volume (**Supplementary Table 5**). Further, the dose reduction of the contralateral organs were positively correlated with L in different distance intervals (P<0.05). Especially, the dose the contralateral optic nerve, temporal lobe and parotid gland showed a dramatic reduction in the distance interval of 1.4-2.1cm (**Figure 3**).

The target coverage of the two groups of plans reached more than 95% of the prescription dose coverage, and the individualized PTV1 was better (Conventional vs. Individualized: 98.19% vs. 99.29%, P< 0.001, **Supplementary Table 6**). Although the target coverage of PTV2 was not statistically significant (P=0.125), it showed an increasing trend (Conventional vs. Individualized: 97.02% vs. 97.16%). The Dmean, D95, and D98 increased, and HI and CI decreased for individualized PTV1, while the Dmean, HI, and CI decreased for PTV2. There was no statistically significant difference in Mu and other indicators (P> 0.05, **Supplementary Table 6**).

After comparison of different treatment planning system (TPS), the following conclusions can be drawn (**Table 4**): (1) under the IMRT treatment mode, the Mu of individualized CTV decreased, the Dmean and V100% of PTV1 increased, and HI and CI decreased; the HI and Dmean of PTV2 decreased; doses of OARs dropped in the temporal lobe, optic chiasm, optic nerve, lens, contralateral inner ear, and brainstem (P< 0.05); (2) under the VMAT treatment mode, the Dmean, V100%, D95, and D98 of PTV1 of individualized CTV increased, while HI and CI decreased; the Dmean and HI decreased for PTV2, while V100%, D95, and CI increased; doses of OARs dropped in spinal cord, contralateral temporal lobe, optic nerve, optic chiasm, parotid gland, pituitary, thyroid gland, lens, and contralateral inner ear (P< 0.05); (3) under the TOMO treatment mode, the Dmean, V100%, D2, D95, and D98 were increased and HI and CI decreased for PTV1 with individualized CTV, while Dmean and CI decreased for PTV2; doses of OARs dropped in contralateral temporal lobe, optic nerve, optic chiasm, pituitary, lens, inner ear, and brainstem (P< 0.05).

**Table 4:** Comparison of targets and organs at risk in three radiotherapy techniques.

Objective	Index	IMRT(Mean ± SD)		P-value	VMAT(Mean ± SD)		P-value	TOMO(Mean ± SD)		P-value
		Conventional	Individualized		Conventional	Individualized		Conventional	Individualized	
Mu		2073.7 ± 383.5	1840.7 ± 251.3	0.010	689.9 ± 62.1	681.5 ± 58.9	0.663	6214.7 ± 710.5	6377.2 ± 832.3	0.688
PTV1	Dmean(cGy)	6883.0 ± 119.5	6937.9 ± 103.0	0.040	6922.0 ± 77.3	6952.0 ± 83.7	0.006	6778.1 ± 70.3	6847.4 ± 86.0	0.000
	V100(%)	98.0 ± 1.7	98.9 ± 1.0	0.012	98.1 ± 1.1	99.1 ± 0.7	0.000	98.4 ± 1.1	99.7 ± 0.4	0.000
	D2(cGy)	7457.3 ± 180.4	7391.4 ± 93.1	0.263	7502.0 ± 77.7	7488.3 ± 66.1	0.675	7423.1 ± 65.8	7461.3 ± 52.0	0.004
	D95(cGy)	6149.9 ± 113.5	6151.1 ± 105.9	1.000	6131.5 ± 82.5	6204.1 ± 72.5	0.001	6007.9 ± 52.6	6083.7 ± 98.0	0.000
	D98(cGy)	6059.8 ± 105.8	6034.1 ± 120.6	0.232	6026.3 ± 89.5	6086.1 ± 60.0	0.002	5911.2 ± 61.6	5987.3 ± 102.8	0.000
	HI	1.219 ± 0.034	1.197 ± 0.020	0.009	1.228 ± 0.028	1.210 ± 0.031	0.000	1.233 ± 0.017	1.220 ± 0.019	0.000
	CI	0.540 ± 0.180	0.451 ± 0.190	0.000	0.488 ± 0.172	0.395 ± 0.164	0.000	0.519 ± 0.131	0.424 ± 0.142	0.000
PTV2	Dmean(cGy)	6158.0 ± 126.4	6061.2 ± 156.0	0.001	6172.4 ± 141.9	6120.1 ± 155.9	0.000	6074.8 ± 129.4	6024.7 ± 151.1	0.000
	V100(%)	97.8 ± 1.7	97.5 ± 1.5	0.940	95.6 ± 0.8	96.3 ± 0.9	0.001	98.0 ± 1.2	97.9 ± 1.3	0.623
	D2(cGy)	7425.5 ± 165.8	7356.0 ± 96.9	0.204	7482.2 ± 79.0	7454.5 ± 64.6	0.099	7386.3 ± 60.7	7401.3 ± 69.4	0.092
	D95(cGy)	5523.1 ± 94.5	5487.7 ± 86.0	0.086	5432.1 ± 36.6	5441.8 ± 24.9	0.021	5411.3 ± 57.6	5386.4 ± 104	0.309
	D98(cGy)	5428.3 ± 94.4	5395.3 ± 92.4	0.052	5333.1 ± 37.5	5343.9 ± 34.9	0.066	5315.8 ± 66.1	5271.6 ± 128.1	0.118
	HI	1.351 ± 0.041	1.331 ± 0.020	0.009	1.375 ± 0.023	1.365 ± 0.023	0.001	1.349 ± 0.027	1.360 ± 0.023	0.103
	CI	0.881 ± 0.018	0.869 ± 0.020	0.073	0.825 ± 0.145	0.827 ± 0.032	0.000	0.876 ± 0.068	0.846 ± 0.045	0.000
Spinal cord	Dmax(cGy)	3338.6 ± 205.1	3270.9 ± 303.3	0.601	3180.8 ± 185.3	3081.9 ± 221.7	0.004	3426.7 ± 448.1	3351.3 ± 502.3	0.278
	D0.03cc(cGy)	3283.6 ± 205.9	3196.3 ± 299.0	0.391	3108.9 ± 183.2	3018.0 ± 175.3	0.008	3350.1 ± 422.0	3290.3 ± 470.6	0.397
Temporal lobes-I	D0.03cc(cGy)	6352.7 ± 519.8	5873.8 ± 1030.1	0.009	6319.6 ± 489.7	6306.1 ± 594.9	0.884	6440.3 ± 515.3	6419.0 ± 556.5	0.293
	Dmax(cGy)	6527.2 ± 503.4	6019.0 ± 952.1	0.004	6523.9 ± 460.9	6474.0 ± 549.0	0.388	6525.9 ± 519.1	6506.1 ± 554.6	0.270
Temporal lobes-C	D0.03cc(cGy)	5905.0 ± 398.6	3909.6 ± 881.7	0.000	5826.1 ± 331.4	4426.8 ± 623.8	0.000	5971.3 ± 297.6	4821.2 ± 691.8	0.000
	Dmax(cGy)	6063.3 ± 360.3	4068.7 ± 920.2	0.000	6015.1 ± 358.9	4704.5 ± 650.2	0.000	6093.1 ± 320.8	4894.6 ± 649.8	0.000
Optic chiasm	D0.03cc(cGy)	4172.2 ± 1463.9	3374.7 ± 1492.5	0.002	4271.3 ± 1248.7	3780.5 ± 1349.1	0.000	4655.1 ± 899.2	4193.0 ± 1083.5	0.001
	Dmax(cGy)	4413.1 ± 1468.0	3610.4 ± 1520.3	0.002	4470.5 ± 1284.6	4013.5 ± 1405.8	0.000	4763.0 ± 952.2	4319.0 ± 1122.6	0.001
Optic nerves-I	D0.03cc(cGy)	4089.2 ± 1582.0	3345.2 ± 1689.8	0.002	4175.0 ± 1647.3	3704.8 ± 1699.9	0.000	4860.0 ± 848.7	4325.6 ± 1196.4	0.001
	Dmax(cGy)	4401.0 ± 1607.1	3649.7 ± 1737.1	0.006	4367.2 ± 1652.8	3894.1 ± 1758.9	0.000	4946.4 ± 866.8	4423.1 ± 1220.7	0.001
Optic nerves-C	D0.03cc(cGy)	3942.5 ± 1536.0	2063.1 ± 838.1	0.000	4031.5 ± 1347.6	2670.7 ± 1232.0	0.000	4789.8 ± 671.2	3369.6 ± 1134.1	0.000
	Dmax(cGy)	4279.4 ± 1563.5	2234.6 ± 874.1	0.000	4246.9 ± 1392.3	2919.7 ± 1308.8	0.000	4888.6 ± 677.9	3503.0 ± 1154.6	0.000
Parotid gland-I	V30(%)	43.3 ± 16.1	42.1 ± 15.7	0.748	41.4 ± 12.8	38.9 ± 12.5	0.008	47.0 ± 15.6	46.0 ± 16.9	0.654
	Dmean(cGy)	3224.4 ± 925.4	3117.4 ± 921.5	0.313	3056.2 ± 755.1	2929.5 ± 669.5	0.001	3274.7 ± 745.0	3229.2 ± 775.2	0.397
Parotid gland-C	V30(%)	37.1 ± 5.3	35.4 ± 7.9	0.601	38.6 ± 6.5	36.6 ± 6.3	0.021	43.9 ± 11.1	44.2 ± 13.6	0.688
	Dmean(cGy)	2895.7 ± 200.0	2752.0 ± 323.4	0.062	2874.7 ± 298.6	2740.6 ± 266.3	0.002	3090.5 ± 533.0	3061.6 ± 602.0	0.713
Thyroid	Dmean(cGy)	3998.9 ± 800.3	3922.1 ± 919.9	0.279	4404.2 ± 634.6	4293.1 ± 630.1	0.004	4203.1 ± 491.0	4196.7 ± 494.7	0.765
Larynx	Dmean(cGy)	3671.6 ± 337.4	3706.2 ± 472.9	0.502	3860.7 ± 302.0	3816.5 ± 354.6	0.638	3753.0 ± 263.8	3730.1 ± 277.3	0.351
Pharyngeal const	Dmean(cGy)	3784.9 ± 353.8	3741.0 ± 476.8	0.911	4079.8 ± 290.0	4126.3 ± 395.9	0.713	3802.4 ± 244.1	3788.0 ± 280.7	0.688
Pituitary	Dmean(cGy)	4679.4 ± 656.6	4575.6 ± 1177.7	0.852	5023.7 ± 699.9	4578.9 ± 735.3	0.000	5173.8 ± 605.7	4780.6 ± 810.7	0.000
	Dmax(cGy)	5557.2 ± 568.6	5545.9 ± 928.7	0.167	5580.4 ± 644.0	5343.0 ± 684.3	0.000	5695.4 ± 604.4	5478.3 ± 793.5	0.007
Oral cavity	Dmean(cGy)	3384.7 ± 252.4	3468.3 ± 333.2	0.086	3479.6 ± 290.7	3411.7 ± 336.8	0.209	3544.5 ± 183.4	3506.0 ± 235.5	0.083
Lens-I	Dmax(cGy)	539.1 ± 167.2	468.4 ± 164.8	0.006	524.3 ± 392.3	405.6 ± 203.1	0.000	468.1 ± 99.5	417.5 ± 112.4	0.000
Lens-C	Dmax(cGy)	515.5 ± 141.8	443.4 ± 136.5	0.003	479.1 ± 179.1	397.7 ± 183.3	0.000	448.7 ± 95.3	377.1 ± 104.0	0.000
Inner ear-I	Dmax(cGy)	6446.2 ± 917.2	6403.9 ± 928.0	0.550	6410.2 ± 926.1	6452.3 ± 883.1	0.343	6276.1 ± 907.8	6230.1 ± 940.6	0.022
	Dmean(cGy)	4266.2 ± 815.4	4138.0 ± 839.2	0.079	4151.2 ± 780.5	4081.9 ± 637.0	0.169	4204.9 ± 784.3	4119.8 ± 822.9	0.045
Inner ear-C	Dmax(cGy)	5518.1 ± 486.5	4402.0 ± 600.9	0.000	5552.9 ± 433.8	4648.7 ± 586.3	0.000	5461.4 ± 432.0	4644.1 ± 683.5	0.000
	Dmean(cGy)	3805.9 ± 433.9	2919.5 ± 359.9	0.000	3605.5 ± 381.3	3190.9 ± 466.9	0.001	3645.0 ± 326.8	3083.1 ± 551.2	0.000
Brain stem	D0.03cc(cGy)	4985.2 ± 398.5	4597.3 ± 350.6	0.001	5010.4 ± 432.0	4950.9 ± 481.0	0.256	5090.1 ± 471.5	4963.7 ± 598.7	0.050
	Dmax(cGy)	5148.7 ± 434.8	4705.9 ± 336.9	0.000	5184.6 ± 506.8	5110.3 ± 520.0	0.379	5150.5 ± 475.5	4957.9 ± 547.3	0.001

I, ipsilateral; C, contralateral.

## Discussion

Our individualized CTV delineation refers to previous experience in reducing the target volume, whether it is based on the invasion risk of NPC (11, 12) or confirmed through pathological examination (10). It has been found that for unilateral NPC, routine prophylactic radiation may not be required for the contralateral parapharyngeal space, cranial foramen, and contralateral pharyngeal recess. According to the study of the T stage and invasion pattern, local recurrence of NPC also follows the gradual infiltration pattern, similar to the primary invasion, usually in the ipsilateral cavernous sinus recurrence (13). Xie et al. (14) also delineated the individualized target area of unilateral NPC, excluding the contralateral parapharyngeal space and skull base channel. The 10-year local recurrence-free survival(LRFS), regional recurrence-free survival (RRFS), and overall survival (OS) reached 96.2%, 90.5%, and 84.7%, respectively, achieving good long-term local control and mild late toxicity. Late toxicity mainly occurred in the ipsilateral organs, and the incidence of hearing loss on the ipsilateral side of the tumor (10/95) was significantly higher than that on the contralateral side of the tumor (1/95).

Accordingly, we designed the individualized CTV delineation for eccentric NPC: the contralateral CTV1 is a 5mm geometrical extension that does not include the entire nasopharyngeal mucosa, and the contralateral CTV2 only includes the pharyngeal recess. Our results showed that compared with conventional CTV, the volume of individualized CTV was significantly decreased (P< 0.05). Individualized CTV reduced the doses delivered to OARs in general, including priority level one protective organs such as the spinal cord, brainstem, optic chiasm, optic nerve, contralateral temporal lobe, as well as the parotid gland, thyroid gland, pituitary, lens, and inner ear. As we know, radiation-induced tissue and nerve damage can cause adverse reactions such as xerostomia, vision loss, and hearing loss. Pituitary dysfunction is also a common complication, which increases in incidence from year to year and is lifelong. In addition, radiation-induced brain injury such as brainstem and temporal lobe can cause symptoms such as disturbance of consciousness, ataxia and epilepsy that can affect the quality of life for patients, and the injury is often irreversible. However, the larynx, Pharyngeal const, and oral cavity did not show significant dose downregulation due to being slightly further away from the scope of CTV reduction. In addition, the individualized CTV delineation also fully met the dose and range of target irradiation, ensuring the safety and efficacy of irradiation. All plans with individualized CTV achieved more than 95% of the target volume covered by the prescribed dose, with individualized PTV1 achieving better target volume coverage.

The prospective study published by Lin et al. (15) omits CTV1 and directly defines CTV2 as GTVnx+entire nasopharyngeal mucosa+8mm+corresponding anatomical structure. The 4-year LRFS and RRFS in this study reached 96.6% and 97.7%, respectively, and all local recurrences were intra-field recurrences, but their CTV delineation still uniformly covered adjacent structures regardless of tumor laterality and tumor stage. However, we discussed the exempted irradiation of the contralateral structure of the nasopharynx for eccentric lesions. Subgroup analysis of the T stage found that individualized CTV delineation of eccentric NPC has less benefit in the T4 stage, due to the limited contralateral structures that can be exempted in high stage patients. A study published this year (16) proposed a concept of CTV delineation including distance and substructure by combining the laterality of tumors and the propagation mode of NPC. The anatomical structures around the nasopharynx were defined as grade 1, grade 2, grade 3, and grade 4, respectively. And the CTV delineation was based on the combination of distance and substructure, When the nasopharyngeal tumor invades different substructures, the corresponding CTV contains different regions. The 5-year LRFS and OS rates for the overall population were 93.2% and 91.5%, respectively. Grade 3 late toxicities included xerostomia, subcutaneous fibrosis, and hearing impairment, with a total incidence of 1.5%. For the eccentric NPC, we also proposed a distance-based concept of delineation, where the contralateral structures is positively correlated at different distance intervals. When the distance between the tumor center and the midline of the body is in the range of 1.4-2.1cm, the individualized CTV delineation would be able to bring greater benefits to organ protection, such as contralateral optic nerve, temporal lobe and parotid gland. This phenomenon may be attributed to the anatomical characteristics of nasopharyngeal carcinoma (NPC), where eccentric tumors predominantly localize in the ipsilateral pharyngeal recess with low contralateral invasion risk (10%). When the tumor is located 1.4-2.1 cm from midline, its distance from the contralateral pharyngeal recess enables more effective sparing of contralateral organs at risk (**Supplementary Figure 1**).

The comparison between VMAT and IMRT in the treatment of NPC found that the target dose of VMAT was higher than that of IMRT, and the maximum dose to the brainstem, spinal cord, and optic nerve, as well as the incidence of late toxic side effects, were lower than those of IMRT (17). However, other studies have shown that IMRT has better conformity than VMAT, similar in the protection of OARs (18), and similar survival outcomes (19, 20). Refined the T stage showed that VMAT provides better OAR retention and HI in the early T stage (21, 22). Du et al. (23) summarized the results of 190 NPC patients treated by TOMO, showing that TOMO had low toxicity and good efficacy. Another study retrospectively compared the efficacy of TOMO and IMRT in the treatment of NPC and found no significant difference in 5-year OS between the two techniques (24). However, TOMO is superior to VMAT in HI and CI (25) and dosimetric parameters of target volume, brainstem, spinal cord, lens, and parotid gland for advanced NPC, and there is no significant difference in survival outcome (26). It can be seen that IMRT, VMAT, and TOMO have similar efficacy, but further studies are needed to determine whether they can effectively reduce the radiation dose to OARs. Our subgroup analysis results show that all three radiotherapy techniques can meet the PTV coverage of treatment needs, but compared with IMRT and VMAT, the prescription dose optimization of PTV1 in TOMO is better, which is consistent with the research conclusion of Chen et al (26). However, under the VMAT treatment mode, the dose of OARs was significantly reduced, including the spinal cord, parotid gland, and thyroid gland, which were not different from IMRT and TOMO. Additionally, VMAT is superior to IMRT in the protection of glands and also associated with lower in MUs and treatment times in this analysis, consistent with previous studies (18, 27). The advantage may stem from the staging of patients and different algorithms employed by the TPS. Variation in TPS results stems partly from different dose calculation algorithms; Eclipse typically uses AAA, while Pinnacle employs the Collapsed Cone Convolution (CCC) algorithm. Although CCC can overestimate dose near bone due to scaling water-derived kernel (28), its convolution/superposition approach generally models lateral electron scatter and energy transport more accurately in heterogeneous regions like the head and neck (with air cavities and bone interfaces) compared to pencil-beam-like algorithms such as AAA (29), which underestimates dose by missing lateral scatter. This superior handling of heterogeneity, combined with Pinnacle’s efficient convolution-based optimization for VMAT, underpins its enhanced performance in NPC planning: the algorithm enables precise dynamic MLC modulation for highly conformal doses around complex, concave targets, significantly improving OAR sparing (e.g., reducing mean parotid dose), while continuous arc optimization minimizes MUs and treatment time, reducing effects like tongue-and-groove. Thus, CCC’s accurate modeling in heterogeneous media and optimized VMAT delivery make it advantageous for NPC.

There are some limitations in our study. First, the radiotherapy plans we have redefined were completed by four senior physicists at our center, and it is still inevitable that there may be some differences in the completion of radiotherapy plans among different physicists. Second, the study of which radiotherapy technique is more appropriate for the individualized CTV of eccentric NPC was based on subgroup analysis, and the exploratory results cannot be directly used for clinical decision-making, and more studies are needed to confirm. While the subgroup analyses in **Table 3** provide preliminary insights into potential effect modifiers, these findings should be interpreted with caution due to the inherent limitations of multiple comparisons. and we acknowledge that formal correction methods were not applied given the exploratory nature of these analyses. Finally, our comparison between conventional and individualized CTV delineation stays in dosimetric analysis without the support of survival data, which is not equivalent to clinical benefits, and the sample size was constrained by clinical availability. More prospective studies are needed to support the translation of the benefits of dose distribution into clinical advantages in reducing radiation toxicity and improving survival. Our center is also conducting a prospective study of the corresponding individualized CTV delineation.

## Conclusion

Individualized CTV delineation for eccentric NPC can achieve significant reduction in the target volume and overall OAR dose, with a significant reduction in contralateral organs, and meet the prescription dose coverage required for treatment. We proposed a distance-based concept for the eccentric NPC, which is easy to be applied in the clinical practice. In addition, subgroup analysis showed that the OAR benefit of individualized CTV was more significant in the VMAT treatment mode. Our prospective trial is currently ongoing for further research on efficacy and feasibility of our individualized CTV method based eccentric distance (NCT06167109).

## Data Availability

The raw data supporting the conclusions of this article will be made available by the authors, without undue reservation.
