# Supplementary material for: Individualization of clinical target volume delineation in eccentric nasopharyngeal carcinoma: a prospective comparative study
Source: Front Oncol. 2025 Aug 4;15:1587764. doi: 10.3389/fonc.2025.1587764 (PMC12358488; doi:10.3389/fonc.2025.1587764)
Supplement: Supplementary file 1 [file DataSheet1.docx]

Supplementary Table 1: Dose restriction on organs at risk

| Structures | Priority level | Dose–volume constraints |
| --- | --- | --- |
| Brain stem | Ⅰ | PRV D0.03cc≤54Gy  Dmax≤54Gy |
| Spinal cord | Ⅰ | PRV D0.03cc≤45Gy  Dmax≤45Gy |
| Optic nerves | Ⅰ | PRV D0.03cc≤54Gy  Dmax≤60Gy |
| Optic chiasm | Ⅰ | PRV D0.03cc≤54Gy  Dmax≤60Gy |
| Temporal lobes | Ⅰ | T1-T2:PRV D0.03cc≤65Gy  T3-T4:PRV D0.03cc≤70Gy  Dmax≤72Gy |
| Lens | Ⅱ | Dmax≤15Gy |
| Pituitary | Ⅱ | Dmax≤65Gy |
| Parotid gland | Ⅲ | Dmean≤26Gy  V30Gy≤50% |
| Larynx | Ⅲ | Dmean≤35Gy |
| Oral cavity | Ⅲ | Dmean≤40Gy |
| Thyroid gland | Ⅲ | Dmean≤45Gy |
| Pharyngeal const | Ⅲ | Dmean≤45Gy |
| Inner ear | Ⅲ | Dmean≤45Gy  Dmax≤55Gy |

Supplementary Table 2: Consistency kappa analysis

|  |  | Expert 2 | | Total | kappa | U | P |
| --- | --- | --- | --- | --- | --- | --- | --- |
|  |  | valid | invalid |  |  |  |  |
| Expert 1 | valid | 16 | 1 | 17 | 0.828 | 3.757 | <0.001 |
|  | invalid | 0 | 3 | 3 |  |  |  |
| Total | | 16 | 4 | 20 |  |  |  |

| Target | Mean±SD | | P-value |
| --- | --- | --- | --- |
|  | Conventional | Individualized |  |
| CTV1(cc) | 95.81±30.53 | 57.57±24.69 | 0.000 |
| CTV2(cc) | 428.50±91.52 | 395.05±92.93 | 0.000 |

Supplementary Table 3: Volume of Conventional and Individualized for CTV

Supplementary Table 4: Dosimetric comparison of Conventional and Individualized for organs at risk

| OAR | Objective | Mean±SD | | P-value |
| --- | --- | --- | --- | --- |
|  |  | Conventional | Individualized |  |
| Spinal cord | Dmax(cGy) | 3311.7±332.2 | 3228.9±386.2 | 0.008 |
|  | D0.03cc(cGy) | 3241.8±318.5 | 3163.7±360.5 | 0.006 |
| Temporal lobes-I | D0.03cc(cGy) | 6373.8±503.6 | 6251.5±726.9 | 0.051 |
|  | Dmax(cGy) | 6525.4±488.0 | 6383.0±684.5 | 0.006 |
| Temporal lobes-C | D0.03cc(cGy) | 5900.1±337.7 | 4461.7±787.4 | 0.000 |
|  | Dmax(cGy) | 6056.2±342.7 | 4633.5±779.3 | 0.000 |
| Optic chiasm | D0.03cc(cGy) | 4397.0±1187.4 | 3847.6±1312.2 | 0.000 |
|  | Dmax(cGy) | 4570.5±1210.2 | 4039.9±1343.7 | 0.000 |
| Optic nerves-I | D0.03cc(cGy) | 4420.2±1404.7 | 3862.9±1554.9 | 0.000 |
|  | Dmax(cGy) | 4598.6±1400.8 | 4042.9±1580.6 | 0.000 |
| Optic nerves-C | D0.03cc(cGy) | 4304.2±1235.4 | 2802.6±1214.5 | 0.000 |
|  | Dmax(cGy) | 4502.2±1240.4 | 2989.3±1248.2 | 0.000 |
| Parotid gland-I | V30(%) | 44.0±14.7 | 42.4±15.2 | 0.123 |
|  | Dmean(cGy) | 3178.8±789.8 | 3088.0±775.4 | 0.002 |
| Parotid gland-C | V30(%) | 40.3±8.8 | 39.3±10.7 | 0.156 |
|  | Dmean(cGy) | 2962.8±400.9 | 2867.2±459.6 | 0.002 |
| Thyroid | Dmean(cGy) | 4234.3±639.1 | 4171.5±669.7 | 0.008 |
| Larynx | Dmean(cGy) | 3776.1±302.2 | 3758.0±357.7 | 0.495 |
| Pharyngeal const | Dmean(cGy) | 3905.6±318.1 | 3908.0±411.8 | 0.942 |
| Pituitary | Dmean(cGy) | 5003.4±674.3 | 4656.0±875.8 | 0.000 |
|  | Dmax(cGy) | 5619.5±608.4 | 5441.3±782.0 | 0.000 |
| Oral cavity | Dmean(cGy) | 3483.1±249.8 | 3460.9±300.1 | 0.441 |
| Lens-I | Dmax(cGy) | 505.9±263.0 | 424.4±164.2 | 0.000 |
| Lens-C | Dmax(cGy) | 475.6±143.7 | 400.1±146.8 | 0.000 |
| Inner ear-I | Dmax(cGy) | 6366.5±909.4 | 6355.4±910.9 | 0.019 |
|  | Dmean(cGy) | 4198.0±782.0 | 4109.2±751.7 | 0.002 |
| Inner ear-C | Dmax(cGy) | 5509.6±442.1 | 4590.8±630.1 | 0.000 |
|  | Dmean(cGy) | 3666.3±377.9 | 3087.5±486.7 | 0.000 |
| Brain stem | D0.03cc(cGy) | 5035.4±437.9 | 4875.4±523.0 | 0.000 |
|  | Dmax(cGy) | 5163.2±474.0 | 4959.4±514.4 | 0.000 |

I, ipsilateral; C, contralateral;

Supplementary Table 5: Correlation analysis results

|  | L | ∆CTV1 | ∆CTV2 |
| --- | --- | --- | --- |
| L | 1 |  |  |
| ∆CTV1 | 0.220* | 1 |  |
| ∆CTV2 | 0.044 | 0.521** | 1 |

*: P＜0.05；**: P＜0.001;

ΔCTV1, ΔCTV2: the difference between the volume of conventional CTV1, CTV2 and individualized CTV1, CTV2.

Supplementary Table 6: Dosimetric comparison of Conventional and Individualized for PTVs

|  | Index | Mean±SD | | P-value |
| --- | --- | --- | --- | --- |
|  |  | Conventional | Individualized |  |
|  | Mu | 3164.4±2532.9 | 3171.3±2645.7 | 0.054 |
| PTV1 | Dmean(cGy) | 6857.5±107.2 | 6908.3±100.9 | 0.000 |
|  | V100(%) | 98.19±1.26 | 99.29±0.77 | 0.000 |
|  | D2(cGy) | 7461.4±110.7 | 7455.8±77.2 | 0.343 |
|  | D95(cGy) | 6087.9±102.8 | 6145.5±104.5 | 0.000 |
|  | D98(cGy) | 5989.4±104.8 | 6036.1±102.3 | 0.000 |
|  | HI | 1.228±0.026 | 1.211±0.025 | 0.000 |
|  | CI | 0.512±0.159 | 0.420±0.162 | 0.000 |
| PTV2 | Dmean(cGy) | 6131.4±139.8 | 6069.8±158.1 | 0.000 |
|  | V100(%) | 97.02±1.64 | 97.16±1.40 | 0.125 |
|  | D2(cGy) | 7432.3±107.6 | 7411.5±83.3 | 0.583 |
|  | D95(cGy) | 5444.7±75.0 | 5430.8±86.6 | 0.555 |
|  | D98(cGy) | 5348.1±78.1 | 5327.6±104.6 | 0.194 |
|  | HI | 1.359±0.031 | 1.355±0.026 | 0.011 |
|  | CI | 0.857±0.103 | 0.844±0.039 | 0.000 |


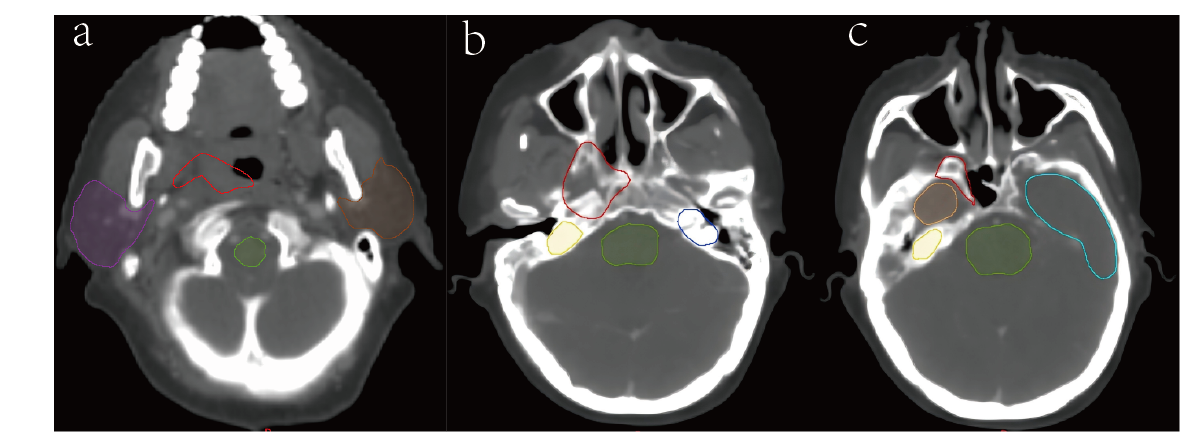


Supplementary Figure 1: A schematic diagram of the spatial relationship between tumor location and important organs at risk. The GTV is in solid red, the brainstem is green, the right parotid gland is purple, the left parotid gland is brown, the right optic nerve is yellow, the left optic nerve is dark blue, the right temporal lobe is orange, and the left temporal lobe is light blue.
